# Cross-Sectional Information on Pore Structure and Element Distribution of Sediment Particles by SEM and EDS

**DOI:** 10.1155/2017/9876935

**Published:** 2017-09-14

**Authors:** Minghong Chen, Huiming Zhao, Hongwei Fang, Yuefeng Zhang

**Affiliations:** ^1^College of Water Resources and Civil Engineering, China Agricultural University, Beijing 100083, China; ^2^State Key Laboratory of Simulation and Regulation of River Basin Water Cycle, China Institute of Water Resources and Hydropower Research, Beijing 100048, China; ^3^Department of Hydraulic Engineering, State Key Laboratory of Hydro-Science and Engineering, Tsinghua University, Beijing 100084, China; ^4^Development Research Center of the Ministry of Water Resources of China, Beijing 100038, China

## Abstract

The interaction between pollutants and sediment particles often occurs on the particle surface, so surface properties directly affect surface reaction. The physical and chemical processes occurring on sediment particle surfaces are microscopic processes and as such need to be studied from a microscopic perspective. In this study, field emission scanning electron microscopy (SEM) and energy dispersive X-ray spectrometer (EDS) were adopted to observe and analyze the pore structure and element distribution of sediment particles. In particular, a special method of sample preparation was used to achieve the corresponding cross-sectional information of sediment particles. Clear images of a particle profile and pore microstructure were obtained by high-resolution SEM, while element distribution maps of sediment particles were obtained by EDS. The results provide an intuitive understanding of the internal microenvironment and external behavior of sediment particles, in addition to revealing a significant role of pore microstructure in the adsorption and desorption of pollutants. Thus, a combination of different experimental instruments and observation methods can provide real images and information on microscopic pore structure and element distribution of sediment particles. These results should help to improve our understanding of sediment dynamics and its environmental effects.

## 1. Introduction

Environmental problems and ecological contradictions are becoming increasingly prominent in the world, and the adsorption and desorption of contaminants onto and from sediment particles have become hot topics in the study of water environment [1–3]. Given that the interaction between contaminants and sediment particles often occurs on the particle surface, the surface properties of particles can directly affect the surface reaction. Compared with studies on geochemical properties, research on the surface properties of sediment particles is limited. Studies to date have focused on change in substance concentration in the solid-liquid interface, but the distribution of the adsorbed matter on the sediment surface and changes in the pore microstructure of sediment particles after adsorption has received little attention. Previous studies have investigated the surface properties of pure materials (especially pure oxide and hydrated oxide), but the properties of natural complex sediment particles are poorly understood. However, information on the pore microstructure and element distribution on the surfaces of sediment particles is necessary to lay a foundation for studying the relationship between sediment particles and pollutants.

Since the physical and chemical processes occurring on the sediment particle surface are microscopic processes, they need to be studied at a microscopic scale. The range of fine sediment size is in microns and millimeters, so the needs of sediment research are difficult to meet by merely observing the fine sediment surface with naked eyes. Thus, surface information of sediment particles should be obtained through experiments and observations. The surface pores of sediment particles significantly influence their physical and chemical properties but are difficult to observe because of their small size. In recent years, more than 60 types of surface analysis methods and analytical instruments have been introduced. Some of the most commonly used microscopic equipment includes scanning electron microscope (SEM), transmission electron microscope (TEM), atomic force microscope (AFM), environmental scanning electron microscope (ESEM), energy dispersive X-ray spectrometer (EDS), energy loss spectrometer (ELS), X-ray diffraction (XRD), and inductively coupled plasma-mass spectrometry (ICP-MS) [4–11]. Among these, high-resolution electron microscopy has been used the most extensively. Electron microscopy can visually display sediment particle morphology and carry out element detection of the sediment particle surface with the addition of a variety of ancillary equipment. Thus, it can be a useful tool to study the interaction between sediment particles and pollutants.

Field emission scanning electron microscopy (FESEM) and energy dispersive X-ray spectrometry (EDS) were adopted in this study to observe and analyze the pore microstructure and element distribution of sediment particles. In particular, a special method of sample preparation was used to obtain the corresponding cross-sectional information. Clear images of particle cross-sectional profiles and pore morphology of sediment particles were provided by high-resolution SEM, and the element distribution maps of sediment particles were obtained by EDS.

## 2. Materials and Methods

### 2.1. Experimental Apparatus

#### 2.1.1. SEM

The main equipment used for microscopic observation was FESEM. The signals most commonly used by SEM are secondary and backscattered electron signals. The former is generally used to display surface topography, and the latter is used to show the atomic number contrast. An S-5500 SEM (JEOL Ltd., Tokyo, Japan) was used in the current study. SEMs with different resolution have different requirements for sample preparation. S-5500 SEM has a high resolution with magnification of 60-2,000,000, and its secondary electron resolution ranges from 0.4 nm (30 kV) to 1.6 nm (1.0 kV). Specific parameters of the electron microscope were selected based on the purpose of the experiment and need for measurement accuracy.

#### 2.1.2. EDS

**Figure 1:**
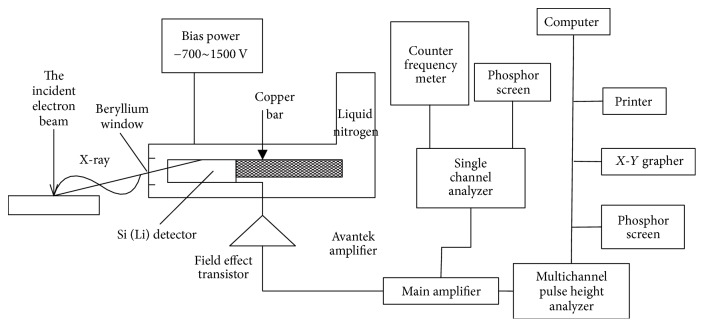
Working principle of EDS.

SEM is equipped with EDS. EDS uses a focused electron beam acting on a small area of the observed sample to excite the characteristic X-ray of the elements contained in the sample. It then captures, processes, and analyzes the information to obtain qualitative or quantitative results of the sample elements.  Figure 1 illustrates the working principle of EDS [12]. EDS can simultaneously accept and detect X-ray photon signals of all energies. Therefore, all the elements in a sample can be analyzed within a few minutes. Given that SEM has high spatial resolution, energy spectrum analysis can be carried out at the micron and submicron scale. In addition, sediment samples need to be coated with a carbon film first and then deposited in the sample chamber to enhance conductivity. The carbon film can cause some interference to light elements (below Be), so a general energy spectrometer was mainly used to analyze the content and distribution of elements with large atomic numbers.

### 2.2. Sample Preparation

**Figure 2:**
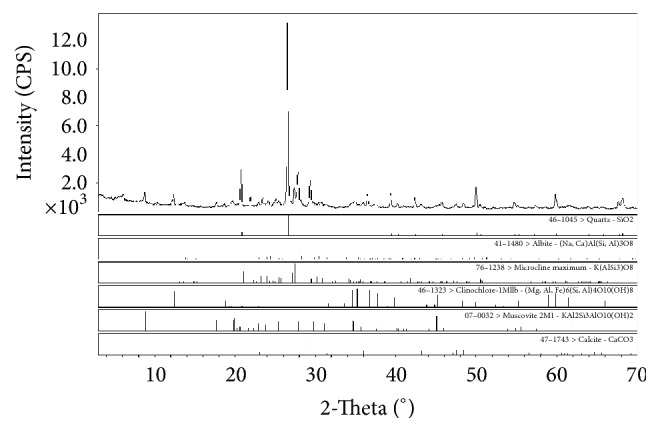
Powder XRD image of sediment.

The sediment used in the experiment was obtained from Guanting Reservoir of Beijing, China. After sampling, it was cleaned, dried, and cleared of impurities and coarse sediments through a 2.0 mm sieve. This sample contained mostly fine particles with particles less than 0.02 mm in size comprising 94.4% of the total. The mineral composition of sediment samples in Guanting Reservoir was basically the same.  Figure 2 shows the XRD energy spectrum graph of these sediment samples. The horizontal axis refers to the measured diffraction angle depending on the cell structure and element position in the crystal system, and the vertical axis represents the relative detection intensity. The mineral compositions were distinguished by a series of characteristic lines corresponding to each mineral. The results revealed that the major components were quartz (38%), followed by albite and muscovite (both 18%). The samples also contained a small amount of microcline (9%), calcite (9%), and clinochlore (8%).

S-5500 SEM is an ultra-high-resolution SEM with high requirements for special sample preparation. Its sample holder is a chamfer box of 1.5 cm × 0.5 cm × 0.4 cm. The thickness of the sample on the sample holder was kept below 2 mm. Given that the sediments were granular materials that failed to meet the requirements of sample preparation, they were mosaicked and the final sample size was ensured not to exceed 3 mm × 3 m × 2 mm. The mixture of sediment and phenolic resin was placed into a mosaic machine, melted under 140°C, applied with high pressure, and recooled. The sediment particles were then embedded in resin and shaped into cylindrical workpieces. The workpieces were ground and polished to smoothen fractures for further analysis. After polishing, the workpiece was cut precisely into 2 mm thick sheet samples by lathe, and the samples were ground into blocks of 3 mm × 3 mm using sandpaper. After ultrasonic cleaning and drying, the sample was glued on the sample holder and then sent into the coater for film coating.

Given that the sediment is nonconductive in a vacuum chamber, no clear image could be obtained from any sediment with no coating. Two kinds of coating apparatus were used in sample preparation, namely, a gold coater and a carbon coater. When we only sought to observe the surface morphology of the particles, the surfaces of the sample were coated with gold film. The obtained particle surface image was clear owing to the strong conductive property of gold film. Moreover, gold film does not affect surface morphology. When surface elements of the particles needed to be probed, the samples were coated with carbon film. The atomic number of gold is high, which means that the coverage of gold film is strong, which can interfere with detection of other elements. By contrast, carbon film meets the requirements of conductivity and does not affect detection of elements other than carbon.

## 3. Results and Discussion

### 3.1. Cross-Sectional Information on Porous Microstructure of Sediment Particles

**Figure 3:**
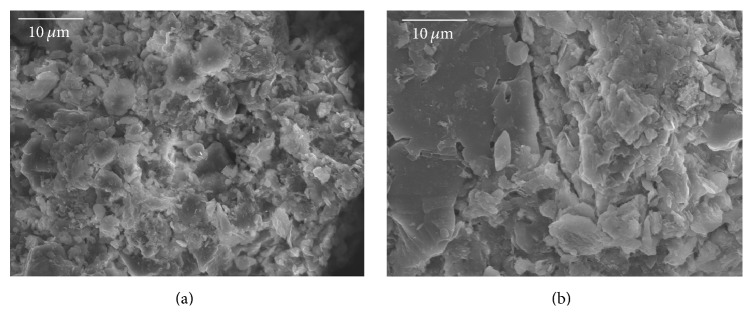
Pores of an individual sediment particle from Guanting Reservoir: (a) Particle 1 (×4000, size: 0.075 mm) and (b) Particle 2 (×8000, size: 0.042 mm).

Pore structure is a common feature of most particulate matter. Previous studies have shown that many pores exist on the surface of sediment particles, which are porous materials [13]. The same conclusion was also drawn by our SEM analysis.  Figure 3 shows the SEM images of natural fine particles from Guanting Reservoir. The images indicate that the pores on the surface of sediment particles were dense and of different sizes. Formation of pores on sediment particle surface is caused by two major factors: the mineral structure of the particles, and the aggregation and bridging of various substances adsorbed on the particle surface. The effects of these two kinds of pores on sediment particles are quite different, and the pore sizes also vary. The former is at the nanoscale, and the latter is in tens or even hundreds of nanometers. As seen in Figure 3, the surface structure of cohesive fine sediment is complex, and the porosity of fine sediments is obviously greater than that of coarse particles. Therefore, cohesive fine sediments exhibit strong adsorption ability.

**Figure 4:**
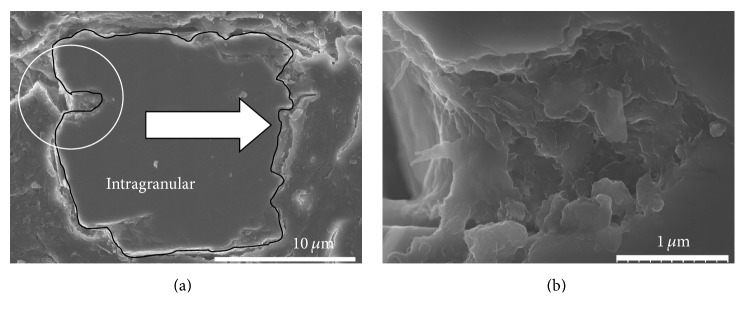
High-resolution SEM images of sediment particle profile and pore microstructure: (a) edge morphology of particle profile (size: 0.017 mm) and (b) amplified pore structure.

To study the pore microstructure of sediment particles, a special mosaic method for sample preparation was used for observation using an ultra-high-resolution electron microscope.  Figure 4 shows the SEM results of a sediment particle from Guanting Reservoir.  Figure 4(a) shows a cross-sectional profile of the particle (marked with a black line). Outside the black line is phenolic resin.  Figure 4(b) is a magnified image of the delineated region in Figure 4(a). Large and small pores could be seen on sediment particles. Most pores were closed, and only a few were connected. Pores were rough and bumpy and contained smaller pores. The sizes of those tiny pores ranged from several nanometers to hundreds of nanometers. It can be inferred from Figure 4 that natural sediment particles have no smooth and spherical surfaces but very complex surface morphologies.

### 3.2. Cross-Sectional Information on Element Distribution of Sediment Particles

**Figure 5:**
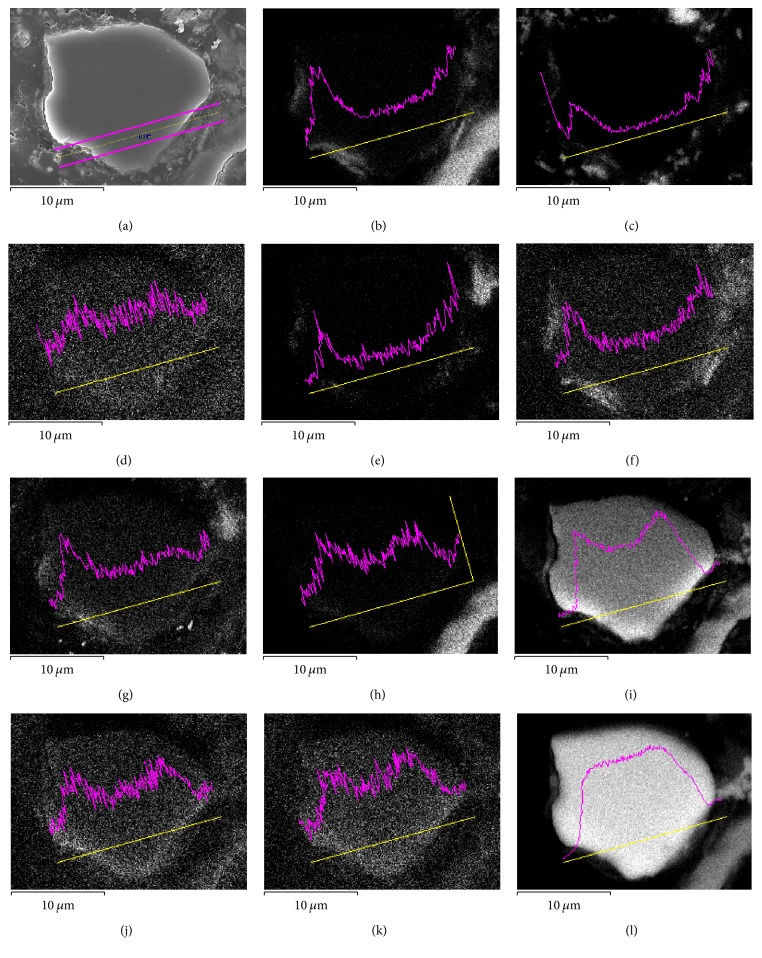
Line scanning results of sediment sample: (a) scanning strip of the sediment particle, (b) Al, (c) Ca, (d) Cl, (e) Fe, (f) K, (g) Mg, (h) Na, (i) O, (j) P, (k) S, and (l) Si.

High-resolution SEM S-5500 with EDS can be used for line scanning of the material surface to analyze the distribution of elements of interest along the scanning line. In this study, the equipment did not scan the surface of sediment particles directly but only one cross section of a sediment particle. Through a special mosaic method for sample preparation, the cross section of a sediment particle embedded in the resin was cut and scanned.  Figure 5 shows the results of line scanning of a sediment sample. The yellow line in Figure 5(a) is the selected scanning strip center line, whereas the two purple thick lines are the scanning strip boundary lines. The purple lines in other subgraphs show the intensity distribution of different elements on the scanning strip.

As shown in Figure 5, the distribution of Si in the particles was relatively uniform, illustrating that the major constituent of sediment particles is SiO_2_. Metal elements of Al, Ca, Fe, K, Mg, and Na formed two peaks, which were seen at the intersection of the scanning line and the particle profile. The location of these peaks suggests that these elements are present in some amount on the surface of the particles. Element O also showed a two-peak distribution, but there is still a certain amount between the two peaks. This finding suggests that numerous oxygen elements existed in the particles, with a large amount distributed on the surface. Thus, most of the surface metal elements existed in the form of oxide (crystal or amorphous) or salt. The distribution peak of element Cl was not obvious and unstable, illustrating that it had very low amount. Elemental P and S were found to be present on the surface of particles, but only in small amount. The distribution map of the line scanning reflects the basic distribution of the elements on the surface of particles.

### 3.3. Observation of Pores and the Porosity of the Sediments

Generally, pores can be classified based on absolute size into supermicropores, ultramicropores, mesopores, and macropores. In addition, they can also be classified by causes and chronological order into primary pores and secondary pores. The pores discussed in this paper are primary intragranular spaces within intact skeletal grains. These pores are generally several to tens of nanometers in size. We tried to observe the three-dimensional microstructure of these pores using high-resolution SEM. Although other tools such as TEM and SPM offer higher resolution and are therefore able to reveal the crystal or atomic structure [14], the resulting images are range limited and non-three-dimensional. In fact, all imaging methods have a characteristic upper and lower limit to their operating magnification. Methods that are optimized to provide information on pores, especially at very small scales, are not the best methods for understanding the composition of the pores in many cases [15]. In addition, it is important to consider how a specific sample preparation fits into the imaging workflow when multiple imaging technologies are employed so as not to interfere with subsequent measurements. Therefore, careful sample preparation, including mosaicking, grounding, polishing, and coating, is crucial for obtaining meaningful results in this study.

Bulk analysis of porosity and pore size distributions in sediments is accomplished by several methods, and each has advantages and disadvantages [16–18]. However, any particular method of observation on sediments detects only a portion of the total pore population as there are always pores that are blocked from view or too small or too large to be appreciated from a particular image or with a particular method [15]. Here we were unable to measure porosity using SEM images because many pores were blocked, and intragranular pores comprised only a fraction of the total pore population. However, we had measured the porosity and pore size distributions of the same sediment samples from Guanting Reservoir by the gas adsorption method. The average pore sizes of those samples were in the range of 2 to 50 nm, with the largest pore volume being 3.7 nm in size [17].

Since pores in the range of 2 nm or smaller overlap with the size of spaces related to crystal site vacancies and other crystal defects, they are not considered to be part of porosity in general. However, this kind of pores is of great significance to the study of adsorption and desorption of pollutants on sediment particles. The pores of sediment particles increase the specific surface area, and large specific surface area greatly enhances the physical and chemical effects on the microinterface (solid-liquid interface). We previously reported that the adsorption of phosphorus onto sediments decreases the volume of pores smaller than 10 nm by filling these pores with contaminants [17]. Luo et al. [19] also found that the fill of Enano-TiO2 particles into the micropores of sediments could significantly reduce t-Plot micropore specific surface area and cause slight decrease in sediment P binding energy.

### 3.4. Evidence of Phosphorus Adsorption on Sediment from Element Distribution

**Figure 6:**
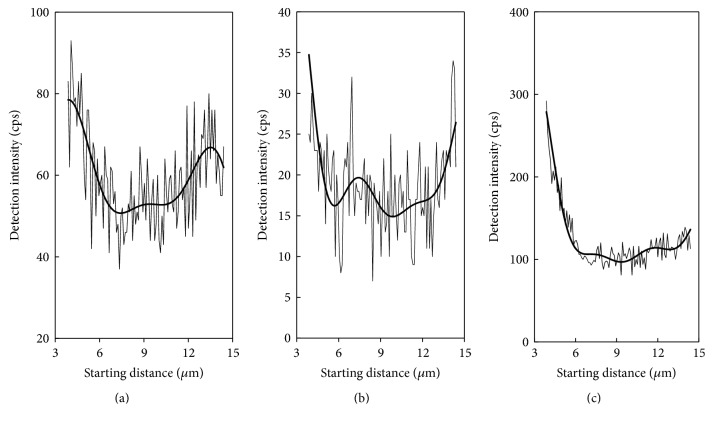
Line scanning energy spectrum of phosphorus: (a) P, (b) Fe, and (c) Al.

Many researchers have reported that the oxides of surface metal elements, especially Al and Fe, are important for adsorption of phosphorus and other pollutants [2, 20–22]. Sediments containing high proportions of Fe or Al oxide minerals have particularly high buffering capacities. The theoretical maximum quantity of P which can be adsorbed in all the pools (fast reversible adsorption to the surface sites plus diffusion limited time-dependent adsorption or deposition below surfaces) is equal to [Al_ox_ + Fe_ox_] [20]. Based on this, we combined our analysis of P, Fe, and Al distribution.  Figure 6 illustrates the line scanning energy spectrum of P, Fe, and Al in Figures 5(j), 5(e), and 5(b). The spectral lines were filtered by high frequency to obtain the black wavy line. The half width of the wave line is related to the width of the scanning stripe and the distribution of elements on the surface of the particle. As the scanning strip intersects the particle profile at 3.8 *μ*m and 14.4 *μ*m, we cut the signals outside of the profile. As seen in Figure 5, elemental P exhibited consistency with elements Fe and Al. Also, it exhibited strong correlation with Fe (*r* = 0.74, *p* < 0.01) and Al (*r* = 0.89, *p* < 0.01), which provided evidence that Fe and Al (hydr)oxide in sediment minerals played important roles in P adsorption.

## 4. Conclusions

SEM and EDS were used to observe the micropore structure and elemental distribution of natural sediment samples after sampling and special treatment. Cross-sectional information on the pore microstructure characteristics and element distribution rule was analyzed in detail. The combination of a variety of experimental instruments and observation methods provided real images and information on the microscopic pore structure and element distribution of sediment particles. Our SEM images showed that complex pore microstructures are an important part of sediment particle morphology. The characteristics of pores have an important influence on the sediment particle microinterface, especially on the adsorption and desorption of pollutants by sediment particles. Our scanning results showed that the metal elements of Al and Fe existed on the surface of the particle, and elemental P exhibited consistency with elemental Fe and Al with correlation of 0.74 and 0.89, respectively. This underscores the importance of Al and Fe (hydr)oxide for P adsorption onto sediment particles. Our findings indicate that observation of the microscopic pore structure and element distribution can lead to a better understanding of the interaction between sediment and pollutants and ultimately of environmental effects of sediments.
